# Efficacy and Safety of Efgartigimod for Patients With Myasthenia Gravis in a Real‐World Cohort of 77 Patients

**DOI:** 10.1111/cns.70391

**Published:** 2025-04-16

**Authors:** Sijia Hao, Zhe Ruan, Rongjing Guo, Qingqing Wang, Xiaoxi Huang, Chao Sun, Huanhuan Li, Ting Gao, Yonglan Tang, Xiangqi Cao, Yu Liu, Zhuyi Li, Ting Chang

**Affiliations:** ^1^ Department of Neurology, Tangdu Hospital The Fourth Military Medical University Xi'an China

**Keywords:** efgartigimod, generalized estimating equation, MG‐ADL, myasthenia gravis, real‐world evidence

## Abstract

**Aims:**

Efgartigimod, a first‐in‐class neonatal Fc receptor antagonist, is approved for generalized myasthenia gravis (gMG). Its safety and efficacy across MG subtypes remain unclear.

**Methods:**

This single‐center real‐world study (September 2023–July 2024) analyzed patients from an MG registry study in China. The primary efficacy outcome is the mean MG‐ADL score changes from baseline at weeks 4, 8, and 12, analyzed via generalized estimating equations. Safety was assessed by adverse events.

**Results:**

Among 77 patients (mean age 56.1 ± 15.2 years; 59.7% male), 76 completed at least one treatment cycle (20 completed 2 cycles; 1 completed 3 cycles). After efgartigimod treatment, MG‐ADL scores decreased significantly by week 4 (mean difference −6.4, 95% CI −7.2 to −5.6, *p* < 0.001), sustaining through week 12 (−6.9, −7.8 to −6.1, *p* < 0.001). After the second cycle, MG‐ADL scores at week 12 trended lower than the first cycle (mean difference: −0.8, 95% CI: −2.0 to −0.5, *p* = 0.061). Efficacy was consistent across MGFA classes and thymoma status. In refractory patients, efgartigimod reduced MG‐ADL scores (*p* < 0.001). Adverse events occurred in 3.9% (3/77).

**Conclusion:**

Efgartigimod safely improved MG‐ADL scores and reduced steroid use across MG subtypes, with sustained efficacy through multiple treatment cycles. These findings support its potential when conventional therapies fail.

## Graphical abstract

This real‐world study of 77 myasthenia gravis (MG) patients treated with efgartigimod showed rapid symptom improvement across disease severities. Sustained efficacy was achieved with repeated cycles, positioning efgartigimod as a promising MG therapy combining swift response and durable maintenance benefits.

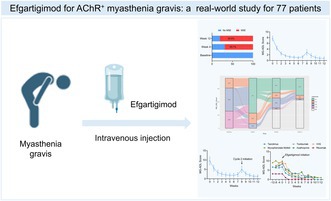

## 1 Introduction

Myasthenia gravis (MG) is a chronic, autoimmune, neuromuscular junction disease caused by impaired neuromuscular junction transmission, affecting eye movement, swallowing, speech, and respiratory function, severely reducing quality of life [1, 2]. Almost 85% of MG patients have acetylcholine receptor (AChR) antibodies [3]. Treatments include glucocorticoids, intravenous immunoglobulin (IVIG), plasma exchange, and immunosuppressants [4, 5]. While effective for most MG patients [6], approximately 10% show poor responses or critical deterioration [7, 8, 9], urgently requiring safer targeted therapies.

FcRn prolongs IgG lifespan, sustaining high serum levels [10, 11]. Efgartigimod is currently the first and only FcRn antagonist that is approved in China for adult patients with AChR‐Antibody^+^ generalized MG (gMG). In Phase I/II trials, efgartigimod significantly reduced concentrations of all IgG subtypes and improved MG symptoms [12, 13]. The global Phase III ADAPT study and Phase III open‐label extension study reported favorable efficacy and safety profiles for efgartigimod in gMG patients [14, 15]. In a multicenter cohort in China, patients with systemic MG or MG crisis also benefited from efgartigimod [16]. As these studies were limited to single‐cycle efgartigimod treatment and lacked detailed patient characteristics, there is a need to evaluate the real‐world impact of efgartigimod in MG.

In this study, we analyzed a real‐world cohort with 77 AChR‐MG patients to investigate the clinical use of efgartigimod in Chinese MG patients. We assessed the efficacy and safety of efgartigimod across different MG subtypes and treatment cycles.

## 2 Methods

### 2.1 Study Population

We retrospectively analyzed AChR‐MG patients treated with efgartigimod at Tangdu Hospital of the Fourth Military Medical University between September 2023–July 2024. Data originating from an MG registry (> 2000 patients) was ethically approved by the Ethics Committee of Tangdu Hospital, Fourth Military Medical University (no. 202102‐06). All clinical data were collected via medical records and follow‐up interviews (in‐person/video) with informed consent.

Inclusion criteria: (1) Clinical manifestations aligning with ocular MG (oMG) or gMG; (2) Confirmed AChR antibody seropositivity; (3) Efgartigimod therapy. Exclusion Criteria: (1) Seropositivity for muscle‐specific kinase (MuSK) or lipoprotein receptor‐related protein 4 (LRP4) antibodies, or triple‐seronegative status; (2) Abnormal repetitive nerve stimulation findings indicative of other neuromuscular junction disorders (e.g., congenital myasthenic syndromes, Lambert‐Eaton myasthenic syndrome); (3) Pregnancy at enrollment or planned pregnancy during the study period.

Clinical status and disease severity were determined by the Myasthenia Gravis Foundation of America (MGFA) and German guidelines. OMG, which corresponds to MGFA I, was defined as symptoms limited to the eye muscles for at least 3 months; gMG, which corresponds to MGFA II, III, IV, and V, was defined as MG symptoms involving limbs, trunk, dysphagia, dysphonia, or respiratory muscles.

### 2.2 Information Collection

Basic information and clinical characteristics of the included patients were collected, including gender, age, disease duration, MGFA classification, patients with thymoma, history of thymectomy, previous treatment with immunotherapies/biologics, the prednisone dosage, and the MG Activity of Daily Living (MG‐ADL) score. Previous treatments include: pyridostigmine, prednisone, immunosuppressants (i.e., azathioprine, tacrolimus, mycophenolate mofetil, cyclosporine, methotrexate and cyclophosphamide), and targeted immunotherapies (i.e., rituximab and tocilizumab).

### 2.3 Treatment and Follow‐Up

All patients received efgartigimod at a dose of 10 mg/kg per dose by intravenous infusion over a period of 1 h, once a week for 4 weeks, as one treatment cycle. Based on the results of the ADAPT NXT (NCT04980495) study and clinical experience, the frequency of efgartigimod treatment for some patients was later adjusted to 10 mg/kg every 2 weeks. Treatment response based on MG‐ADL scores [17] and safety profiles were recorded weekly for 12 weeks after the initiation of efgartigimod treatment. We conducted standardized video‐based or in‐person clinical assessments using a unified case report form to collect MG‐ADL scores and therapeutic feedback. To minimize potential bias introduced by remote evaluation, all assessments were performed by the same neurologist, who administered an identical questionnaire protocol to that used during in‐person consultations. Changes in prednisone dosage were recorded before starting exemplary and at the end of each dose. Clinically meaningful improvement (CMI) was defined as a ≥ 2 points reduction in MG‐ADL score from baseline values. Minimal symptomatic expression (MSE) was defined as an MG‐ADL score of 0 or 1 [18].

### 2.4 Statistical Analysis

Continuous variables were expressed as mean ± standard deviation (SD). We conducted normality tests on all continuous variables using the Shapiro–Wilk test. Comparisons between two time points were analyzed using a paired *t*‐test or Wilcoxon signed‐rank test. Comparisons between more than two time points were analyzed by generalized estimating equations (GEE) analysis. Categorical data were expressed as percentages (%) and compared by Fisher's exact test. *p* < 0.05 was considered statistically significant, and all tests were two‐sided. GraphPad Prism (version 8.4.2) and SPSS 25.0 were used.

## 3 Results

### 3.1 Basic Characteristics of the Study Population

From September 2023 to July 2024, a total of 77 patients with MG were administered efgartigimod, and the basic characteristics were shown in Table 1. A total of 46 males and 31 females were included with an average age of 56.1 ± 15.2 years. All MG patients were AChR antibody seropositive. The mean duration of disease in these included patients was 3.6 ± 3.8 years. Twenty‐four patients had thymoma. Of these, 21 patients underwent thymectomy before efgartigimod injection, and 3 patients underwent thymectomy after the administration of efgartigimod. The baseline IgG levels were 12.0 ± 5.8 g/L.

**TABLE 1:** Basic demographics and clinical characteristics of MG patients with efgartigimod treatment.

Clinical variables	AchR‐MG patients (*n* = 77)
Age (mean ± SD) years	56.1 ± 15.2
Sex (%)	
Male	46 (59.7%)
Female	31 (40.3%)
Duration of MG, years	3.6 ± 3.8
Thymoma (%)	24 (31.2)
Thymectomy before efgartigimod	21 (27.3)
Previous treatments	
Pyridostigmine	77 (100%)
Prednisone	48 (62.3%)
Tocilizumab	2 (2.6%)
Tacrolimus	13 (16.9%)
IVIG	2 (2.6%)
Mycophenolate Mofetil	5 (6.5%)
Azathioprine	3 (3.9%)
Rituximab	1 (1.3%)
Treatments with efgartigimod	
Prednisone	48 (62.3%)
Tacrolimus	8 (10.4%)
Mycophenolate Mofetil	4 (5.2%)
Rituximab	1 (1.3%)
MG‐ADL at baseline (mean ± SD)	7.7 ± 3.6
MGFA (%)	
I	9 (11.7%)
II	29 (37.7%)
III	34 (44.2%)
IV	5 (6.4%)
Prednisone dosage at baseline (mg/day)	26.3 ± 13.3
IgG level at baseline (g/L)	12.0 ± 5.8

All patients received pyridostigmine before the efgartigimod injection. Previous treatments included prednisone (48 of 77, 62.3%), tacrolimus (13 of 77, 16.9%), azathioprine (3 of 77, 3.9%), mycophenolate mofetil (5 of 77, 6.5%), targeted immunotherapies such as rituximab (1 of 77, 1.3%), tocilizumab (2 of 77, 2.6%) and IVIG (2 of 77, 2.6%). The MGFA classification at the time of onset includes MGFA I (9 of 77, 11.7%), MGFA II (29 of 77, 37.7%), MGFA III (34 of 77, 44.2%), and MGFA IV (5 of 77, 6.4%).

Of these 77 included patients, 76 patients received at least one cycle of efgartigimod, and 1 patient only received two injections of efgartigimod due to side effects. A total of 20 patients received 2 cycles of efgartigimod treatment, and 1 patient received 3 cycles of efgartigimod. Among these patients, 6 patients received efgartigimod treatment every 2 weeks after the previous treatment cycle. The main reasons for initiating efgartigimod included a poor response to previous treatment, contraindications to other treatments, and the need for rapid improvement of MG symptoms.

### 3.2 Efficacy of Efgartigimod Treatment

**FIGURE 1:**
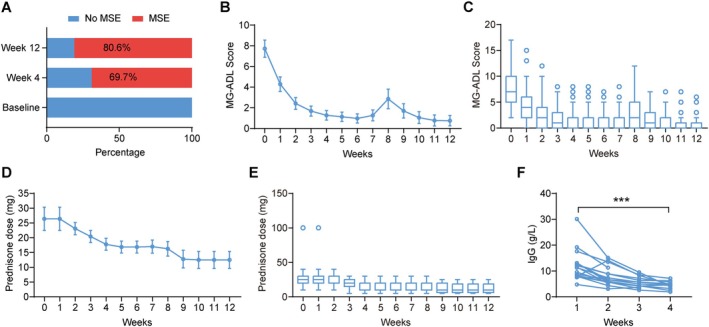
The efficacy of efgartigimod (at least one cycle) in MG patients. (A) The percentage of MG patients with MSE at baseline, weeks 4, and 12 after efgartigimod treatment. (B, D) Mean changes and 95% confidence interval of MG‐ADL score (B) and daily prednisone dose (D) in MG patients after efgartigimod treatment for at least one cycle. (C, E) Box plots show the changes of MG‐ADL score (C) and daily prednisone dose (E) in MG patients after efgartigimod treatment for at least one cycle. (F) The IgG levels of MG patients after efgartigimod treatment for at least one cycle. MG‐ADL, myasthenia gravis activities of daily living. ***p < 0.001.

Among the 76 patients receiving efgartigimod for at least one cycle, all had improvement in clinical symptoms. In the entire cohort, the baseline MG‐ADL was 7.7 ± 3.6 before efgartigimod treatment. After efgartigimod treatment, 98.7% of patients (75 of 76) rapidly achieved CMI with a mean time of 1.3 ± 0.7 weeks. After 4 weeks, 69.7% of cases (53 of 76) achieved MSE (Figure 1A), while the MG‐ADL scores decreased to 1.3 ± 2.0. As compared to baseline, the MG‐ADL scores at week 4 were significantly reduced (mean difference: −6.4; 95% confidence interval [CI]: −7.2 to −5.6, *p* < 0.001. Table 2, Figure 1B,C). This significant difference was sustained until week 12 (mean difference: −6.9, 95% CI: −7.8 to −6.1, *p* < 0.001. Table 2, Figure 1B,C). At week 12, 80.6% of cases (29 out of 36) achieved MSE (Figure 1A).

**TABLE 2:** Efficacy for patients with efgartigimod treatment (at least one cycle) by generalized estimating equation analysis.

Outcome	Patients (*n* = 76)	Time effect	Mean difference^a^ (95% CI)	*p*
MG‐ADL score, mean (95% CI)				
Baseline	7.7 (6.9–8.5)	< 0.001	—	—
Week 4	1.3 (0.8–1.7)		−6.4 (−7.2 to −5.6)	< 0.001
Week 8	2.9 (1.9–3.8)		−4.9 (−5.9 to −3.8)	< 0.001
Week 12	0.8 (0.3–1.3)		−6.9 (−7.8 to −6.1)	< 0.001
Daily prednisone dose, mg, mean (95 CI%)				
Baseline	26.4 (22.6–30.2)	< 0.001	—	—
Week 4	17.8 (15.8–19.7)		−8.6 (−12.0 to −5.2)	< 0.001
Week 8	16.3 (13.9–18.6)		−10.1 (−13.6 to −6.6)	< 0.001
Week 12	12.5 (9.9–15.1)		−13.9 (−17.6 to −10.2)	< 0.001

^a^ Versus baseline.

In 47 patients who received at least one cycle of efgartigimod and prednisone, the average baseline dose of prednisone was 26.4 ± 13.4 mg. After the use of efgartigimod, the average daily dose of prednisone was significantly reduced at week 4 (mean difference: −8.6, 95% CI: −12.0 to −5.2, *p* < 0.001) and sustained until week 12 (mean difference: −13.9, 95% CI: −17.6 to −10.2, *p* < 0.001. Table 2, Figure 1D,E). Among the 47 patients, 27.7% (13 of 47) received a daily dose of prednisone ≤ 10 mg in the fourth week. At week 12, 55.0% of patients (11 of 20) were taking prednisone doses of ≤ 10 mg per day. Moreover, 74.5% were able to reduce their daily dose of prednisone by week 4. After the initiation of efgartigimod therapy, some patients received additional immunotherapy, including tacrolimus (8 patients), rituximab (1 patient), and mycophenolate mofetil (4 patients).

In 18 patients, we measured the total IgG levels in blood samples collected until the end of one cycle. The baseline of total IgG level in 18 patients was 12.0 ± 5.8 g/L. At the end of the first cycle, the total IgG level of 18 patients was 4.5 ± 1.6 g/L, with a reduction of 62.5% (Figure 1F).

**FIGURE 2:**
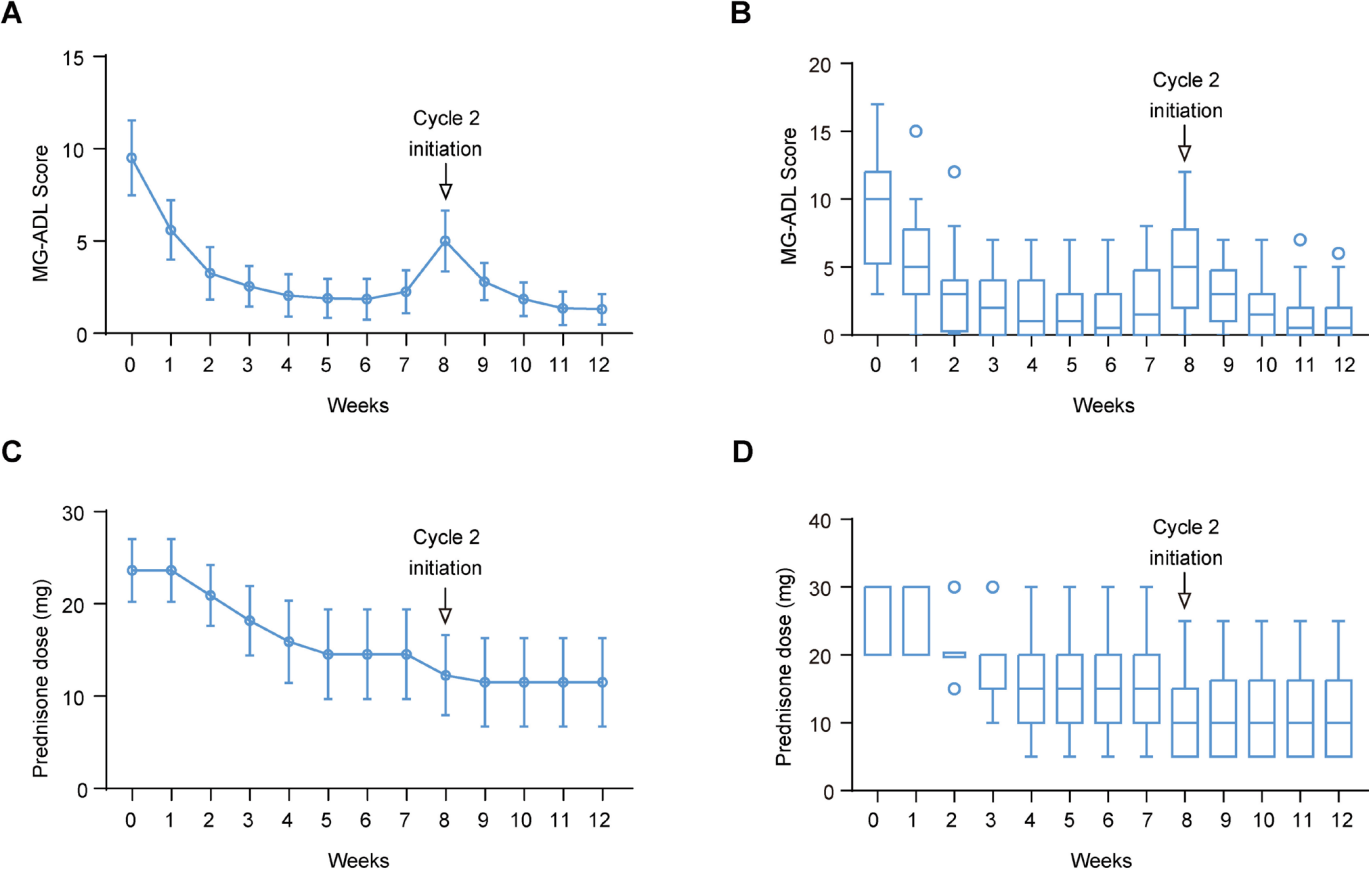
The efficacy of efgartigimod in MG patients receiving two cycles. (A, C) Curve plot shows the mean changes and 95% confidence interval of MG‐ADL score (A) and daily prednisone dose (C) in MG patients after efgartigimod treatment with two cycles. (B, D) Box plot shows the changes of MG‐ADL score (B) and daily prednisone dose (D) in MG patients after efgartigimod treatment with two cycles.

26.3% of patients (20 of 76) started a second cycle of efgartigimod therapy with a mean interval of 37 days. Among the 20 patients who received 2 cycles of efgartigimod therapy, 75.0% (15 of 20) initiated cycle 2 because of symptom fluctuations, with the longest time between two cycles being 157 days for one patient. Among these 20 patients, the baseline MG‐ADL was 9.5 ± 4.3 and decreased to 2.1 ± 2.4 after 4 weeks (mean difference: −7.5, 95% CI: −9.1 to −5.8, *p* < 0.001, Table S1, Figure 2A,B). However, the mean MG‐ADL value was increased to 5.0 ± 3.5 after 8 weeks (mean difference: 2.9, 95% CI: 1.3–4.7, *p* < 0.001, Table S1, Figure 2A,B). After the second cycle of efgartigimod treatment, the MG‐ADL was decreased to 1.3 ± 1.8 points by week 12 and lower than the MG‐ADL after the first cycle of treatment (mean difference: −0.8, 95% CI: −2.0 to −0.5, *p* = 0.061, Table S1, Figure 2A,B), and 65% of patients (13 of 20) achieved MSE.

The average baseline dose of prednisone was 23.6 ± 5.0 mg in 11 patients receiving two cycles of efgartigimod and prednisone. The use of efgartigimod reduced the mean daily dose of prednisone to 15.9 ± 6.6 mg at week 4 (mean difference: −7.7, 95% CI: −10.6 to −4.8, *p* < 0.001). At week 12, the mean daily dose of prednisone was reduced to 11.5 ± 6.7 mg, with a decrease in the mean daily dose of 12.1 ± 2.7 mg (mean difference: −12.1, 95% CI: −16.3 to −8.0, *p* < 0.001, Table S1, Figure 2C,D). 3 of 11 patients (27.3%) received a prednisone dose ≤ 10 mg at week 4. Among all 11 patients receiving prednisone at baseline, 81.8% reduced their daily dose of prednisone at week 4. Moreover, the average daily dose of prednisone did not fluctuate when symptoms fluctuated at week 8, and the lower dose of prednisone was maintained during cycle 2 treatment of efgartigimod.

### 3.3 Efficacy of Efgartigimod in MG Patients With Different MGFA Classifications and Clinical Severity

The mean time to reach CMI after efgartigimod treatment was 1.7 ± 1.1, 1.3 ± 0.7, 1.2 ± 0.5, and 1.2 ± 0.4 weeks for patients with MGFA I, II, III, and IV, respectively. Although more severe, MGFA III and IV patients achieved CMI in a shorter period. Among the 9 MGFA I patients (oMG patients), 77.8% of patients (7 of 9) achieved CMI. The baseline MG‐ADL score was 4.0 ± 1.5, and after 4 weeks, the MG‐ADL score was decreased to 1.6 ± 2.1 (mean difference: −2.4, 95% CI: −3.5 to −1.4, *p* < 0.001, Table S2). At week 12, the MG‐ADL score was 0.3 ± 0.5 (mean difference: −3.8, 95% CI: −4.8 to −2.7, *p* < 0.001, Table S2), and all these patients achieved MSE. These results demonstrate the efficacy of efgartigimod in oMG patients.

For patients with MGFA II, the proportion of patients reaching MSE after 4 weeks was 89.7%, suggesting the rapid onset of efficacy of efgartigimod treatment in patients with mild‐state MG. For patients with more severe MGFA III and IV, baseline MG‐ADL scores were 10.3 ± 2.5 and 12.8 ± 3.3, respectively. After 4 weeks, the MG‐ADL scores were decreased to 2.0 ± 2.5 (mean difference: −8.2, 95% CI: −9.2 to −7.3, *p* < 0.001, Table S2) and 1.0 ± 1.4 (mean difference: −11.8, 95% CI: −15.5 to −8.1, *p* < 0.001, Table S2), respectively. The proportions of patients reaching MSE by week 12 were 70.6% and 75.0%, respectively.

**FIGURE 3:**
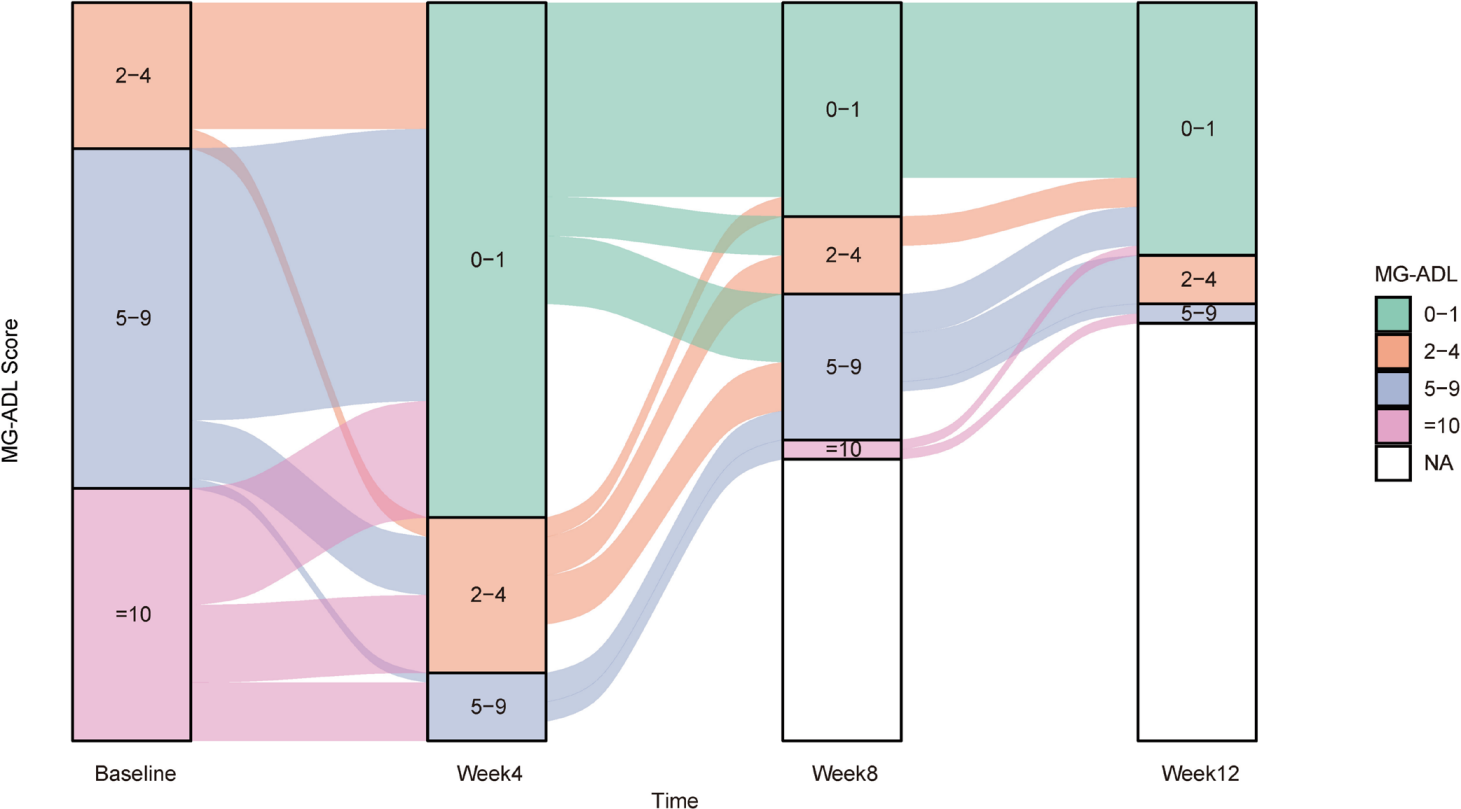
The proportion of MG patients with different MG‐ADL scores. The proportion of MG patients with different MG‐ADL scores at baseline, weeks 4, 8, and 12 after efgartigimod initiation.

We also stratified the clinical severity of patients based on baseline MG‐ADL scores. The proportion of MG patients with high clinical severity (MG‐ADL ≥ 10) decreased from 33.7% at baseline to 0.0% at weeks 4 and 12, respectively. The proportion of MG cases with MSE status increased from 0% at baseline to 69.7% at week 4 and 80.6% by week 12 (Figure 3). Of all patients with the baseline MG‐ADL ≥ 10, approximately 76.9% were in a mild disease state at week 4, i.e., MG‐ADL ≤ 4, and approximately 88.9% were in a mild disease state at week 12.

### 3.4 Efficacy of Efgartigimod in MG Patients With Thymoma

**FIGURE 4:**
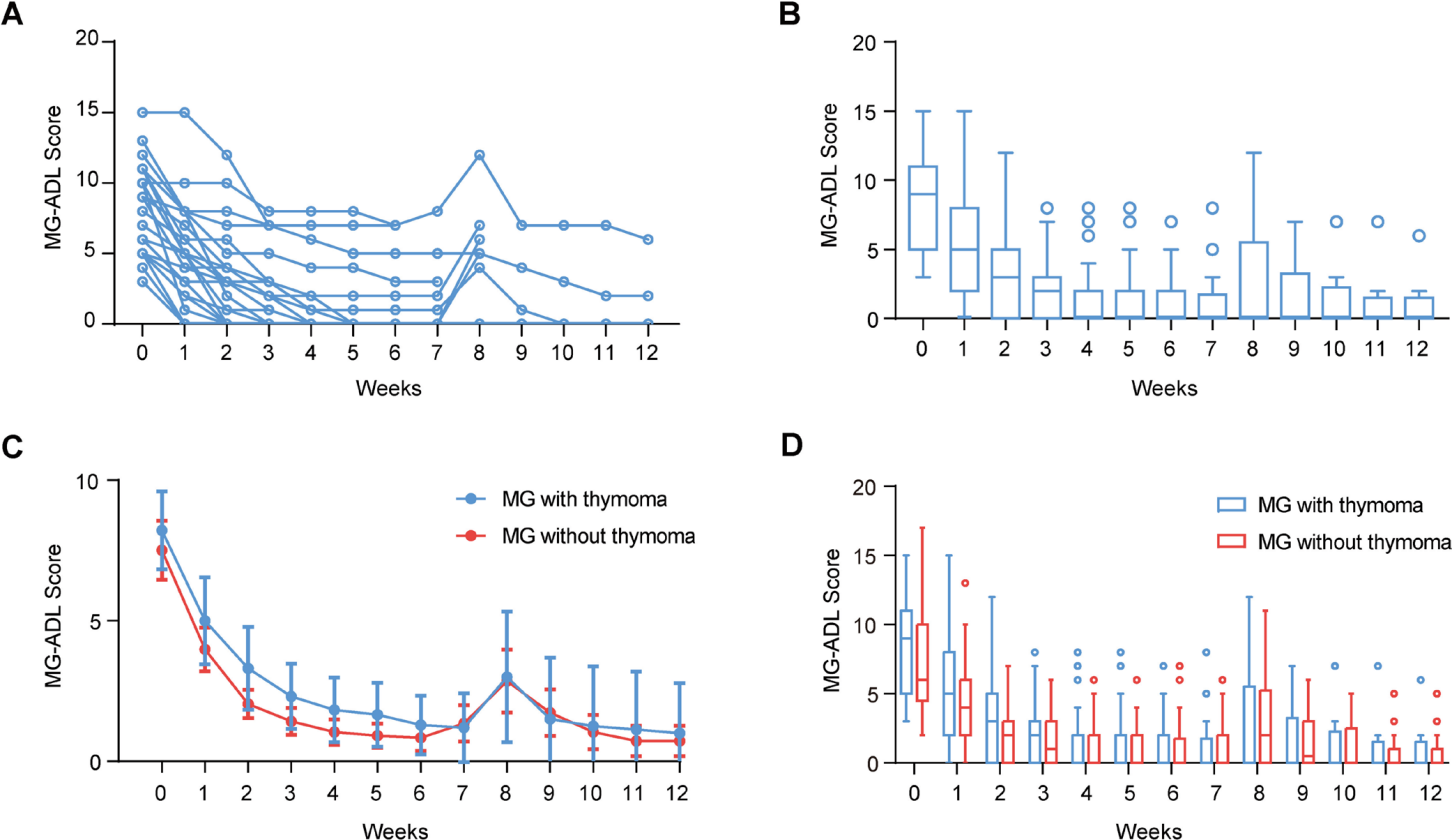
The efficacy of efgartigimod in MG patients with thymoma. (A, B) Curve plot (A) and box plot (B) showed the changes of MG‐ADL score in MG patients with thymoma after efgartigimod treatment. (C) The comparison of MG‐ADL score changes in MG patients with or without thymoma after efgartigimod treatment. Data points show mean scores and error bars show 95% confidence interval. (D) Box plots show the comparison of MG‐ADL score in MG patients with or without thymoma after efgartigimod treatment.

MG patients often have thymoma, but the efficacy of efgartigimod in MG patients with thymoma versus those without thymoma remains elusive. In this cohort, 23 MG patients with at least one cycle of efgartigimod treatment had thymoma. As compared to the MG‐ADL score at baseline, there was a greater decrease in the MG‐ADL score at week 4 (mean difference: −6.4, 95% CI: −7.4 to −5.4, *p* < 0.001). This decrease remained until week 12 (mean difference: −7.2, 95% CI: −8.4 to −6.1, *p* < 0.001, Table S3, Figure 4A,B). The effects of efgartigimod on the MG‐ADL score were similar in patients with thymoma versus those without thymoma (Table S4, Figure 4C,D), suggesting that efgartigimod was effective in MG patients with or without thymoma.

### 3.5 Efficacy of Different Dosing Frequency of Efgartigimod in MG Patients

In the ADAPT NXT study (NCT04980495), patients in the Q2W continuous group (starting with one time/7 days, four consecutive doses, and then 1 time at 1‐week intervals) had less fluctuation of MG‐ADL scores as compared to fixed‐cycle therapy, indicating that Q2W continuous dosing provides more stability. In our cohort, 6 patients received the Q2W dosing regimen. Among these patients, two patients started with a week interval of dosing after 1 cycle, 3 patients started with a week interval of dosing after 2 cycles, and 1 patient started with a week interval of dosing after 3 cycles (Figure S1). We found that, in patients 1 and 2, the MD‐ADL scores tended to be more stable (Patient 1: 3.1 ± 3.2 before Q2W and 0.8 ± 0.9 after Q2W, Patient 2: 1.6 ± 2.4 before Q2W and 0.6 ± 1.1 after Q2W). In contrast, no significant difference after Q2W treatment was observed in other patients.

### 3.6 Efficacy of Efgartigimod Versus Other Immunologic Agents in Patients With MG

Poor response to the previous treatment could be a reason for some patients to use efgartigimod. In 26 patients with a history of using other immunosuppressants or targeted biologics, we found higher MG‐ADL scores (*p* < 0.05), whereas there was a substantial decrease in MG‐ADL scores after initiating efgartigimod treatment (*p* < 0.001, Figure S2A,B). Previously, treatments in these 26 patients included tocilizumab (*n* = 2), tacrolimus (*n* = 13), IVIG (*n* = 2), mycophenolate mofetil (*n* = 5), azathioprine (*n* = 3), and rituximab (*n* = 1). The magnitude of change in MG‐ADL scores was small regardless of the types of immunosuppressants or targeted biologic therapies before efgartigimod. The MG‐ADL scores all decreased after the initiation of efgartigimod treatment (Figure S2C). These results suggest that efgartigimod is effective in MG patients with a poor response to conventional therapy.

### 3.7 Safety of Efgartigimod

Of the 77 patients treated with efgartigimod, 3 patients (3.9%) reported mild to moderate adverse events. The predominant adverse events of the two patients were upper respiratory tract infections, which were resolved with the use of appropriate antimicrobials on an outpatient basis. One patient reported a bilateral lower extremity rash following the administration of efgartigimod, resulting in treatment discontinuation of the application. No adverse events, such as long‐term disability and MG progression, were noted.

## 4 Discussion

This study provides an up‐to‐date analysis of efgartigimod in a real‐world cohort of adult MG patients following its approval in China. These MG patients included a variety of disease types, including oMG patients that were not included in the ADAPT and ADAPT^+^ studies. This study also assessed the efficacy of efgartigimod in MG patients with different severities of MG classified by MGFA criteria at onset. These results suggest that in patients with more severe MG, efgartigimod treatment can rapidly relieve symptoms and maintain long‐term efficacy, which is consistent with the results of the ADAPT study [14]. We evaluated the treatment effects in patients receiving repeated treatment cycles of efgartigimod and compared the efficacy of efgartigimod with other immunosuppressants or targeted biologics in patients with MG.

Emerging biologics like efgartigimod enable targeted immunotherapy with faster action and fewer side effects compared to conventional therapies [19, 20]. In a multicenter study of 14 Chinese centers, about 3.7% of the patients with MG received efgartigimod and had an effective response [16]. Different from this study [16], our study included a larger number of MG cases (77 vs. 61) and patients with oMG. The treatment response of patients in our study is better than in the previous study, i.e., the proportion of cases achieving CMI after one cycle of efgartigimod treatment (97.4% vs. 72%) and the proportion of cases achieving MSE after one cycle of efgartigimod (69.7% vs. 45.9%). The possible reason for this discrepancy is that patients with MG crises were not included in this study. In addition, our cohort had a shorter duration from disease onset to the initiation of efgartigimod (3.6 ± 3.8 years) as compared to the previous studies [7, 14, 16, 21, 22], which may contribute to a better response to efgartigimod, suggesting that the early administration of efgartigimod will be worth studying to provide better benefits for MG patients.

Different from the phase III ADAPT trial and other cohort studies [7, 14], we included patients with oMG in this study. The current treatment for patients with oMG mainly relies on the early use of prednisone and other immunosuppressive agents to reduce the symptoms and prevent the generalization of oMG [23, 24]. To date, there are no clinical data from RCTs on the use of efgartigimod in patients with oMG. In this study, 9 patients received efgartigimod because they could not tolerate the side effects of steroids or other immunosuppressants. In these patients, we found that efgartigimod induced a good response in oMG. To our knowledge, the anatomical specificity of extraocular muscles (simpler NMJ structure, lower AChR density, and reduced complement regulators) may explain oMG's rapid response to efgartigimod's IgG‐reducing mechanism [25]. The rapid decrease in AChR antibody titers following efgartigimod infusion prevents membrane attack complex (MAC) formation and allows for structure repair in EOMs, leading to clinical improvement. These findings suggest that efgartigimod can effectively control oMG, although future larger clinical studies are needed to verify these findings.

We also demonstrated that while efgartigimod therapy showed clinical efficacy across different MGFA classifications of MG patients, patients showed differential therapeutic sensitivities based on disease severity. Notably, patients with higher MGFA classifications (III and IV), despite presenting more severe symptoms, achieved CMI earlier and exhibited greater short‐term reductions in MG‐ADL scores following treatment. This observation can be explained through the analysis of AchR‐MG pathology. The disease mechanism primarily involves pathogenic IgG autoantibodies binding to AChRs, triggering MAC formation that ultimately leads to postsynaptic membrane destruction, characterized by simplified postjunctional folds, reduced AChR density, and synaptic debris accumulation [26, 27]. Moreover, clinical severity directly correlates with autoantibody load in MG patients [28]. Therefore, we hypothesize that the enhanced response in higher MGFA classes may correlate with greater baseline pathogenic IgG levels, wherein efgartigimod's IgG reduction induces more pronounced clinical improvement—a hypothesis requiring validation through serial IgG monitoring.

In this study, we also assessed the effects of different treatment cycles and dosing frequency of efgartigimod on the stability of MG. A portion of patients experienced fluctuations after one cycle of treatment, which was consistent with the previous ADAPT NXT study (NCT04980495). We found that the changes in MG‐ADL total score fluctuated less in patients in the Q2W sustained dosing group, indicating that Q2W sustained dosing was effective in preventing MG recurrence and providing stability. Our use of the dosing strategy to Q2W at a later stage for some patients with fluctuating MG was based on the standard dosing strategy. In two of these patients, we found that the fluctuation of MG‐ADL scores was reduced after the implementation of the Q2W dosing regimen, and the conditions of these patients were more stable. Therefore, for MG patients with fluctuating symptoms after cyclic therapy, the Q2W regimen is more effective in controlling the disease. Due to the small number of cases, further subsequent studies with larger sample sizes are still needed.

MG patients currently have multiple therapeutic options of immunosuppressants, including tacrolimus, mycophenolate mofetil, and the targeted biologics Tocilizumab and Rituximab [29]. Although these treatments have demonstrated their clinical efficacy, many MG patients respond poorly to these treatments [30, 31, 32]. In this study, we found that efgartigimod can provide good efficacy in patients with refractory MG, regardless of prior drug use. Nevertheless, the distinct mechanisms of action between efgartigimod and other biologics retain the potential for combination therapeutic strategies. For instance, rituximab, an anti‐CD20 monoclonal antibody mediating B‐cell depletion, demonstrates established efficacy in MG symptom management [32]. When considering long‐term control, sequential administration or combination regimens integrating rituximab with efgartigimod may offer enhanced therapeutic sustainability. However, this hypothesis requires systematic clinical validation to establish optimal treatment protocols and safety profiles.

Additionally, our study revealed that MG patients without thymoma demonstrated superior clinical responses to efgartigimod therapy compared to thymoma‐associated MG cases, although the intergroup difference did not reach statistical significance. Previous investigations have documented that the thymoma microenvironment facilitates excessive generation and immune escape of Th cells, which subsequently promotes peripheral B‐cell production of AChR‐targeting autoantibodies. Once established, these pathogenic AChR/autoantibody complexes residing in regional lymph nodes may perpetuate myasthenic manifestations, even following thymectomy [33, 34]. This persistent autoimmune activation likely underlies the relatively diminished therapeutic sensitivity to efgartigimod observed in thymoma‐associated MG patients. Given these pathophysiological considerations, future studies employing larger cohorts and extended treatment cycles should be conducted to evaluate efgartigimod's efficacy in this specific MG subtype.

This study also has several limitations. (1) The short follow‐up duration precludes conclusions about long‐term outcomes following efgartigimod administration. (2) Most patients received only one treatment cycle, with limited representation of those undergoing two or three cycles. Future studies should prioritize extended treatment cycles and larger cohorts to better evaluate the efficacy and safety of multi‐cycle efgartigimod regimens. (3) Due to clinical practice constraints, weekly IgG level measurements were unavailable for all patients, limiting our ability to establish robust correlations between efgartigimod use and IgG dynamics. (4) The study exclusively enrolled AChR‐MG patients. Further research is warranted to explore efgartigimod's applicability in other MG subtypes, including MuSK, LRP4, and triple‐negative patients.

## 5 Conclusion

This study provides novel data on the efficacy, safety, and tolerability of efgartigimod in a single‐center, real‐world cohort in China. The use of efgartigimod with a variety of dosing regimens and conditions suggests the reliability of efgartigimod in different types of Chinese MG patients. Future studies with larger sample sizes are required to optimize the indications and dosing of efgartigimod.

## Author Contributions

Ting Chang conceptualized the study, secured funding, and designed uniform procedures for data collection across study centers. Ting Chang, Sijia Hao, Yu Liu, and Zhuyi Li contributed to the study design. Ting Chang, Sijia Hao, Zhe Ruan, and Rongjing Guo contributed to the data analysis and curation. Ting Chang, Sijia Hao, Zhe Ruan, and Rongjing Guo contributed to data interpretation. Qingqing Wang, Xiaoxi Huang, Chao Sun, Huanhuan Li, Ting Gao, Yonglan Tang, and Xiangqi Cao contributed to the data collection. All authors contributed to the article and approved the submitted version.

## Ethics Statement

The clinical data were based on patients from an MG registry study in China that covered more than 2000 patients with MG, was approved by the Ethics Committee of Tangdu Hospital, Fourth Military Medical University (202102‐06).

## Consent

All clinical data were collected after signing an informed consent form.

## Conflicts of Interest

The authors declare no conflicts of interest.

## Supporting information


Data S1.

## Data Availability

The datasets, which include individual participant data and a data dictionary defining each field in the set used or analyzed during the current study, will be available upon reasonable request. Requests for data should be submitted via email to changting1981@163.com.
