# Supplementary material for: Efficacy and Safety of Efgartigimod for Patients With Myasthenia Gravis in a Real‐World Cohort of 77 Patients
Source: CNS Neurosci Ther. 2025 Apr 16;31(4):e70391. doi: 10.1111/cns.70391 (PMC12001068; doi:10.1111/cns.70391)
Supplement: Supplementary file 1 — Data S1. [file CNS-31-e70391-s001.pdf]

Supplementary information

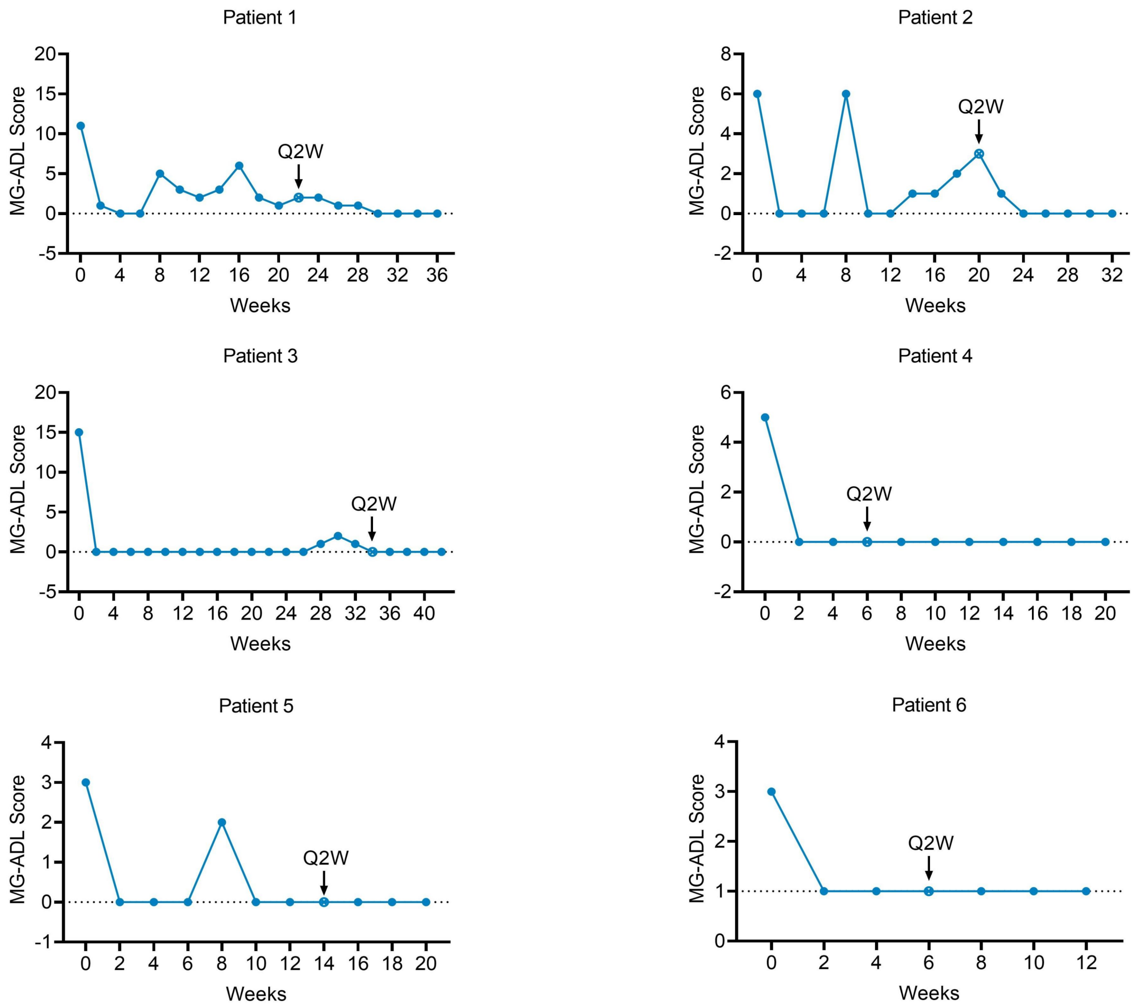

Figure S1. The MG-ADL scores in patients receiving Q2W treatment after efgartigimod initiation.

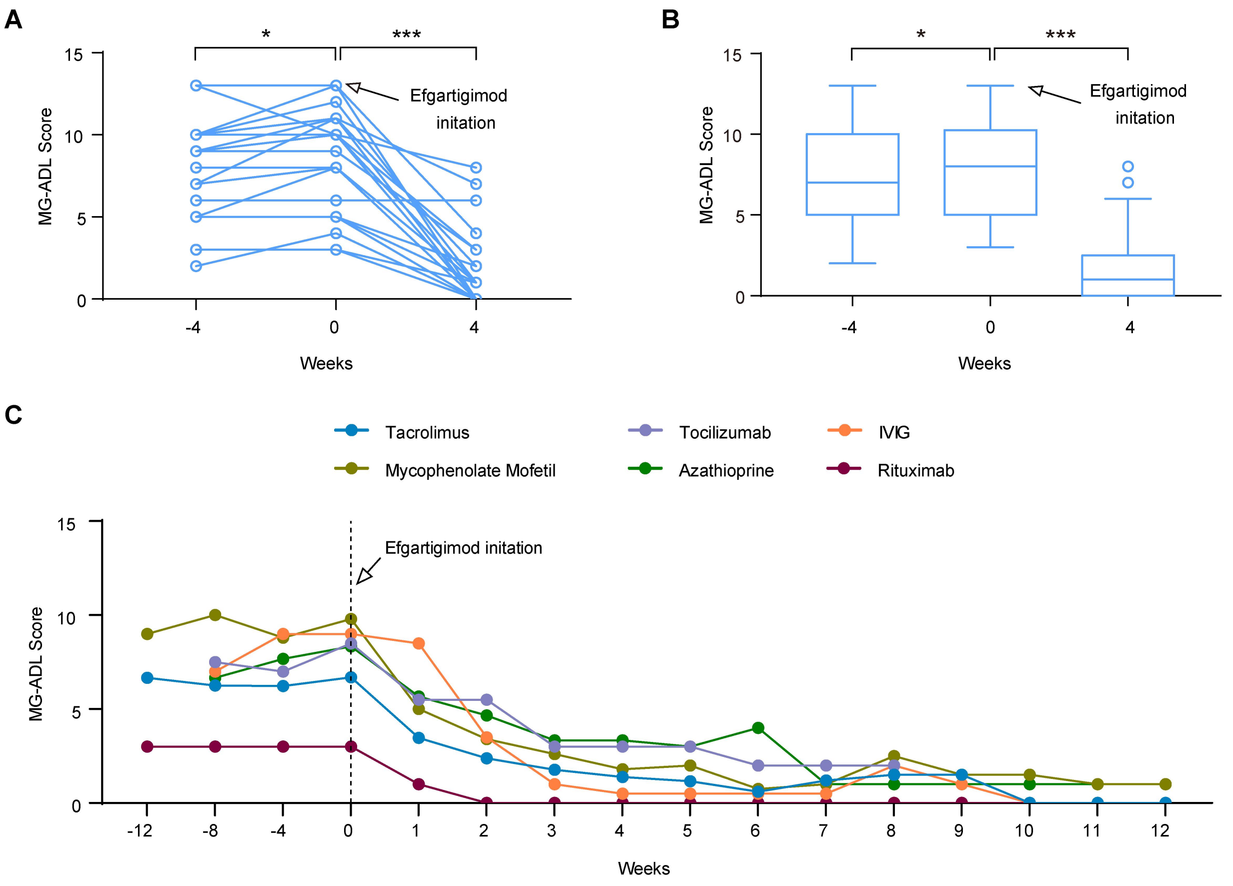

Figure S2. Comparison of the efficacy of efgartigimod versus other drugs in MG patients.

(**A**) Curve plot shows the mean changes and 95% confidence interval of MG-ADL scores in MG patients before and after efgartigimod initiation. (**B**) Box plot shows the changes of MG-ADL scores in MG patients before and after efgartigimod initiation. (**C**) The changes of MG-ADL scores in MG patients treated with tacrolimus, tocilizumab, IVIG, mycophenolate mofetil, azathioprine and rituximab before efgartigimod initiation. * P < 0.05, *** P < 0.001.

Table S1. Efficacy for patients with efgartigimod treatment for two cycles by generalized estimated equation analysis.

Outcome	Patients (n=20)	Time effect	Mean difference (95% CI)	*P*-Value
MG-ADL score, mean (95% CI)				
Baseline	9.5 (7.7 to 11.4)	< 0.001	-	-
Week 4	2.1 (1.0 to 3.1)		-7.5 (-9.1 to -5.8)	< 0.001
Week 8	5.0 (3.5 to 6.5)		-4.5 (-6.4 to -2.6) [a]	< 0.001
			2.9 (1.3 to 4.7) [b]	< 0.001
Week 12	1.3 (0.5 to 2.1)		-8.2 (-9.8 to -6.6) [a]	< 0.001
			-0.8 (-2.0 to -0.5) [b]	0.61
			-3.7 (-5.6 to -1.8) [c]	< 0.001
**Outcome**	**Patients (n=11)**	**Time effect**	**Mean difference[a] (95% CI)**	***P*-Value**
Daily prednisone dose, mg, mean (95 CI%)				
Baseline	23.6 (20.8 to 26.5)	< 0.001	-	-
Week 4	15.9 (12.2 to 19.7)		-7.7 (-10.6 to -4.8)	< 0.001

Week 8	12.3 (8.6 to 15.9)	-11.4 (-15.2 to -7.6)	< 0.001
Week 12	11.5 (7.6 to 15.4)	-12.1 (-16.3 to -8.0)	< 0.001

[a] versus baseline, [b] versus week 4, [c] versus week 8.

Table S2. The change of MG-ADL score for patients with different MGFA classifications after efgartigimod treatment (one cycle at least) by generalized estimated equation analysis.

MGFA	MG-ADL Score	Time effect	Mean difference[a] (95% CI)	*P*-Value
MGFA I (n = 9)				
Baseline	4.0 (3.1 to 4.9)	< 0.001	-	-
Week 4	1.6 (0.2 to 2.9)		-2.4 (-3.5 to -1.4)	< 0.001
Week 8	2.4 (1.1 to 3.8)		-1.6 (-2.5 to -0.6)	0.001
Week 12	0.3 (-0.2 to 0.7)		-3.8 (-4.8 to -2.7)	< 0.001
MGFA II (n = 29)				
Baseline	5.1 (4.5 to 5.7)	< 0.001	-	-
Week 4	0.4 (0.1 to 0.6)		-4.7 (-5.3 to -4.2)	< 0.001
Week 8	2.0 (0.5 to 3.5)		-3.1 (-4.7 to -1.5)	< 0.001
Week 12	0.3 (0.2 to 0.8)		-4.8 (-5.6 to -4.1)	< 0.001
MGFA III (n = 33)				
Baseline	10.3 (9.4 to 11.1)	< 0.001	-	-
Week 4	2.0 (1.2 to 2.9)		-8.2 (-9.2 to -7.3)	< 0.001
Week 8	3.3 (1.7 to 4.9)		-7.0 (-8.6 to -5.3)	< 0.001
Week 12	1.2 (0.3 to 2.0)		-9.1 (-10.1 to -8.1)	< 0.001
MGFA IV (n = 5)				
Baseline	12.8 (10.2 to 15.4)	< 0.001	-	-
Week 4	1.0 (-0.1 to 2.1)		-11.8 (-15.5 to -8.1)	< 0.001

Week 8	3.8 (1.1 to 6.5)	-9.0 (-12.3 to -5.7)	< 0.001
Week 12	0.8 (-0.1 to 1.6)	-12.1 (-15.2 to -8.9)	< 0.001

[a] versus baseline.

Table S3. Efficacy for patients with thymoma after efgartigimod treatment (at least one cycle) by generalized estimated equation analysis.

Outcome	Patients (n=23)	Time effect	Mean difference[a] (95% CI)	*P*-Value
MG-ADL score, mean (95% CI)				
Baseline	8.2 (6.9 to 9.5)	< 0.001	-	-
Week 4	1.8 (0.8 to 2.9)		-6.4 (-7.4 to -5.4)	< 0.001
Week 8	3.0 (1.0 to 5.0)		-5.2 (-7.0 to -3.4)	< 0.001
Week 12	1.0 (-0.4 to 2.4)		-7.2 (-8.4 to -6.1)	< 0.001

[a] versus baseline.

Table S4. Comparison of Efficacy in MG patients with or without thymoma after efgartigimod treatment (at least one cycle) by generalized estimated equation analysis.

**Outcome**	**MG with thymoma (n=23)**	**MG without thymoma (n=53)**	**Time effect**	**Group effect**	**Mean difference[a] (95% CI)**	***P*-Value**
MG-ADL score, mean (95% CI)						
Baseline	8.1 (7.4 to 8.8)	7.7 (7.2 to 8.3)	< 0.001	0.146	-	-
Week 4	1.7 (0.9 to 2.5)	1.2 (0.7 to 1.8)			-0.1 (-1.5 to 1.3)	0.420
Week 8	3.2 (1.5 to 4.9)	2.7 (1.6 to 3.8)			-0.1 (-2.3 to 2.1)	0.934
Week 12	1.2 (0.4 to 1.9)	0.1 (-0.7 to 0.9)			-0.6 (-2.3 to 0.9)	0.911

[a] versus MG patients with or without thymoma.
